# Beneficial effects of communicating intentions when delivering moral criticism: Cognitive and neural responses

**DOI:** 10.3758/s13415-024-01164-1

**Published:** 2024-02-14

**Authors:** Inga K. Rösler, Félice van Nunspeet, Naomi Ellemers

**Affiliations:** 1 Department of Social Psychology, University of Amsterdam, Nieuwe Achtergracht129-B, 1018 WS, Amsterdam, The Netherlands; 2 Utrecht University, Utrecht, The Netherlands

**Keywords:** Feedback, Event-related potential, Morality, Intentions, Intergroup

## Abstract

People often do not accept criticism on their morality, especially when delivered by outgroup members. In two preregistered studies, we investigated whether people become more receptive to such negative feedback when feedback senders communicate their intention to help. Participants received negative feedback from ostensible others on their selfish (rather than altruistic) decisions in a donation task. We manipulated the identity of a feedback sender (ingroup vs. outgroup) and the intention that they provided for giving feedback. A sender either did not communicate any intentions, indicated the intention to help the feedback receiver improve, or communicated the intention to show moral superiority. We measured participants’ self-reported responses to the feedback (Study 1, *N* = 44) and additionally recorded an EEG in Study 2 (*N* = 34). Results showed that when no intentions were communicated, participants assumed worse intentions from outgroup senders than ingroup senders (Study 1). However, group membership had no significant effect once feedback senders made their intentions explicit. Moreover, across studies, when feedback senders communicated their intention to help, participants perceived feedback as less unfair compared with when senders tried to convey their moral superiority. Complementing these results, exploratory event-related potential results of Study 2 suggested that communicating the intention to help reduced participants’ attentional vigilance toward negative feedback messages on their morality (i.e., decreased P200 amplitudes). These results demonstrate the beneficial effects of communicating the intention to help when one tries to encourage others’ moral growth through criticism.

**Supplementary Information:**

The online version contains supplementary material available at 10.3758/s13415-024-01164-1.

“Now if arguments were in themselves enough to make men good, they would […] have won very great rewards […]; but as things are, […] they are not able to encourage the many to nobility and goodness.” (EN X.9. 1179b4–10)

People often try to motivate others to act more morally by criticizing them. However, already around 350 years BC the ancient philosopher Aristotle called into question whether telling someone to act morally is a successful strategy to motivate them to become a good person. In line with this argument, research has demonstrated that when people attempt to encourage others to show moral behavior by criticizing them, this often backfires (Gausel & Leach, 2011; Rösler et al., 2021, 2023; Täuber et al., 2018; Täuber & van Zomeren, 2013). It often makes people defensive, angry, and annoyed. Part of this defensive reaction toward moral criticism might entail that the person being criticized makes assumptions about the underlying motives of the critic. Even though critics often have a specific intention or goal in mind when delivering criticism, they often do not communicate these to the person they are criticizing, leaving room for assumptions.

When the critic’s intentions are unknown, the criticized person might use the group membership of the critic as a cue to *infer* their intentions (Hornsey et al., 2004; Tajfel & Turner, 1979). This is problematic because when a critic is perceived as an outgroup member, people infer more hostile intentions and are less willing to accept such feedback compared with when a critic is an ingroup member (Esposo et al., 2013; Hornsey & Imani, 2004; Hornsey et al., 2004; Thürmer et al., 2019). Thus, being an outgroup member and criticizing others to promote behavioral change can be challenging.

It is important to address these challenges, because criticism of people’s moral behavior coming from outgroup members can offer important clues about where groupthink and group conformity have led to an unethical group culture. It provides a more objective outsider view in situations where ingroup members are afraid to speak up about morally questionable group behaviors for fear of being ostracized (Cavazza et al., 2014; Ellemers & van Nunspeet, 2020; Thau et al., 2015; Van der Lee et al., 2017). More broadly, addressing these challenges could help to improve intergroup contact in times of polarization and “othering” by removing obstacles to successful intergroup communication (Finkel et al., 2020).

## Obstacles to effectiveness of outgroup criticism on peoples’ morality

The first obstacle that both outgroup and ingroup members face when delivering moral criticism is related to what the message entails for the person receiving the criticism. Being criticized about one’s morality means that a central part of the identity of a person is under attack (Aquino & Reed, 2002; Brambilla & Leach, 2014; Pagliaro et al., 2016; Strohminger & Nichols, 2014). Such threat to the self-image often causes people to defend themselves, for example, by assuming that critics have hostile intentions or by getting upset (Gausel & Leach, 2011; Rösler et al., 2021; Täuber & van Zomeren, 2013; Van der Lee et al., 2016). Furthermore, they may engage in coping mechanisms, such as justifying and misremembering their moral shortcomings or emphasizing having changed since their moral failure (Carlson et al., 2020; Kouchaki & Gino, 2016; Mazar et al., 2008; Shalvi et al., 2015; Stanley et al., 2019). Thus, criticizing others for their moral behavior often is ineffective, because people deceive themselves about their moral shortcomings to save face.

The second obstacle outgroup members face when delivering criticism on people’s morality is related to them being perceived as belonging to a different social group by the person they criticize (an outgroup; Ellemers, 2012; Tajfel & Turner, 1979). Social-psychological research has shown that criticism from outgroups often is not accepted (intergroup sensitivity effect; Hornsey & Imani, 2004; Hornsey et al., 2002). Receivers of such criticism may assume that outgroup critics are driven by hostile motives and perceive the criticism as less constructive than that of ingroup critics (Hornsey & Imani, 2004). In the intergroup sensitivity literature, it has been long debated whether this negativity toward outgroup criticism stems from defending the ingroup (Hornsey & Esposo, 2009; Hornsey & Imani, 2004) or is based on a conversational norm proscribing intergroup criticism (Adelman & Verkuyten, 2020; Sutton et al., 2006; Thürmer & McCrea, 2021). Regardless of whether a critical remark is disregarded because of social identity concerns or conversational norms (both processes may play a role), the underlying psychological mechanism of why it is rejected has consistently been attributed to the perceived unconstructiveness of the comment, thus the underlying motive of why criticism was delivered (Adelman & Verkuyten, 2020; Hornsey & Imani, 2004; Thürmer & McCrea, 2018, 2021). This makes it difficult to change people’s perception of outgroup criticism; it is rejected regardless of the quality of the criticism (Esposo et al., 2013). Outgroup critics may even be confronted with hostile responses when they criticize other groups (Thürmer et al., 2019; Thürmer & McCrea, 2018, 2023).

Whereas previous research on the intergroup sensitivity effect has focused mostly on intergroup criticism (e.g., a British citizen criticizing Australians, Hornsey & Imani, 2004), recent studies demonstrate that outgroup criticism is similarly ineffective on an interpersonal level, such as when the self (i.e., an individual’s behavior), rather than an ingroup, is criticized (Rösler et al., 2021, 2023). For instance, people accept and change their behavior less often based on critical comments at the workplace when these are delivered by an outgroup sender than ingroup sender (Rösler et al., 2021). This effect is the result of increased negative perception of the senders’ intentions and a decreased motivation to improve based on criticism, thus showing a similar underlying psychological mechanism as to why outgroup criticism is rejected in intergroup situations. Moreover, experimental research shows that people are more emotionally affected by, thus care more about, receiving criticism from ingroup members than outgroup members in the actual moment (Rösler et al., 2023).

These studies illustrate the challenges that outgroup critics encounter when attempting to encourage personal growth. Recent research in the intergroup sensitivity literature has indicated a promising approach for mitigating negativity toward criticism from outgroup senders, which involves fellow ingroup members actively embracing criticism from an outgroup (i.e., from a vaccine expert; McCrea & Thürmer, 2023). Nevertheless, this strategy may not always be a practical or viable option. Other research has shown that negativity can be mitigated when outgroup critics acknowledge their possession of similar negative traits as those being subjected to criticism (Hornsey et al., 2008). This acknowledgment serves to signal nonhostile motives and fosters a sense of common ground among group members. Establishing common ground may be similarly successful in interpersonal situations, when one’s individual behavior, rather than one’s group identity, is criticized. One way to establish such common ground may be to explicitly confirm that motives are benevolent.

The third obstacle outgroup critics face is related to the type of assumptions that people may make when being criticized on their morality. As previously mentioned, receiving moral criticism often causes people to engage in coping mechanisms, such as making negative assumptions about the intentions of critics. People may assume that outgroup critics want to enhance themselves by criticizing them (downward comparison; Festinger, 1954; Wills, 1981) rather than delivering constructive criticism. For instance, they may assume that the actual intention of outgroup critics when delivering moral criticism is to demonstrate their own moral superiority (Minson & Monin, 2011; Monin et al., 2008). This will make the criticism be perceived as an attack, rather than an opportunity to improve moral behavior. Demonstrating moral superiority often is found to annoy the people who are targeted or witness such behavior (Cramwinckel et al., 2013; Minson & Monin, 2011; Monin et al., 2008). Thus, besides facing obstacles related to the criticism addressing people’s morality and being perceived as an outgroup member, outgroup critics may additionally be confronted with defensive or aggressive responses by the people they criticize because of being accused of wanting to demonstrate moral superiority.

To address these three obstacles, the current research tested whether confirming that outgroup senders have benevolent, rather than harmful, motives for delivering moral criticism can increase the receipt of such criticism.

## Current research

In the current research, we examined whether having outgroup critics communicate their helpful intentions when delivering moral criticism can decrease recipients’ defensiveness toward the feedback. We hypothesized that when outgroup critics communicate *why* they deliver criticism this prevents criticism receivers from making negative assumptions about the critic’s intentions and consequently will improve the receipt of their criticism. Moreover, we hypothesized that criticism receivers would be less defensive toward moral criticism when critics communicate their intention to help by encouraging moral growth, rather than not making intentions explicit or demonstrating moral superiority.^1^

We tested these hypotheses with two experimental studies in which we delivered criticism to participants in the form of negative feedback messages that related to their moral character, following open science practices, such as preregistration and data sharing (also see McCrea et al., 2022; Thürmer & McCrea, 2021). By experimentally manipulating the underlying psychological mechanism derived from previous literature, that is the benevolence of motives, this approach allowed us to make causal inferences about reactions to outgroup criticism in interpersonal settings. To measure participants’ defensive responses, we asked participants to rate the perceived fairness of feedback. Feedback messages were the same across conditions. This implies that instances where participants perceive feedback as more unfair—such as when feedback is delivered by outgroup members rather than ingroup members—may be indicative of defensive responses elicited by our experimental manipulation (other research measured defensiveness with similar items, e.g., perceived credibility or constructiveness; Hornsey et al., 2004; Rösler et al., 2021).

In both studies, we asked participants to perform a donation task where they made repeated monetary allocation decisions. Because the main focus of the current research was to investigate responses to negative feedback on people’s morality, we examined decisions where people made selfish (rather than altruistic) decisions in the donation task. After each decision, they received feedback in the form of trait evaluations of their character (paradigm adapted from Schindler & Kissler, 2018) from ostensible senders who were presented as ingroup or as outgroup members judging participants’ behavioral decisions on the task. Most importantly, the feedback senders’ intentions with delivering a certain feedback message also were presented to participants. Senders either communicated the intention to help the participant improve, to emphasize their own moral superiority, or did not communicate any intentions. We predicted that participants would make more negative assumptions about the intentions of outgroup than ingroup members but that this effect would be attenuated when group members made their intentions explicit. Similarly, we predicted that participants would perceive negative moral feedback as more unfair when outgroup senders than ingroup senders delivered feedback but that this effect of group-membership would also be attenuated when senders made their intentions explicit. Finally, we predicted that participants would perceive negative feedback on their selfish decisions as less unfair when senders communicated their intention to help rather than their moral superiority or did not communicate intentions.

Study 1 was conducted online, measuring participants’ self-reported perceived fairness of the feedback they received. However, self-reports do not always reveal participants’ actual thoughts and feelings, as participants may not be able or willing to accurately report their experiences (Ellemers & van Nunspeet, 2020). This is especially a concern for studying moral criticism situations. Because these situations have a high potential for reputational harm, they may easily be affected by social desirability bias. In Study 2, we therefore additionally examined whether and how participants’ brain activity was affected when receiving feedback. To this end, Study 2 was conducted in the lab, and we recorded an electroencephalography (EEG) to measure participants’ brain responses while they received feedback in the experimental task. More information about the event-related brain potentials is provided in the introduction to Study 2.

## Study 1

### Method

#### Experimental design

The experiment had the following design: 2: Sender’s group-membership (ingroup vs. outgroup, within-participant factor) x 2: Communicated intentions (helping vs. superiority intention, within-participant factor) x 3: Timepoint of communicated intention (before the sender is presented vs. before the feedback message is presented vs. control [no intentions communicated], between-participants factor). The sender’s group-membership was specified to the participant as the ingroup having a similar Social Value Orientation (SVO; Messick & McClintock, 1968; Van Lange, 1999) type as themselves and the outgroup as having a different SVO-type as themselves (without telling participants their own or the outgroup’s type, also see “Procedure, instruments, and stimuli”). This manipulation was established with the SVO-task in which participants were asked to make monetary distribution choices between the self and unknown others. We included the factor “timepoint of communicated intention” to check whether communicating intentions before or after the feedback sender is presented influences the perceived fairness of their feedback. The factor “communicated intentions” was only manipulated within the two conditions that presented intentions to participants and not in the control condition. The valence of the feedback was dependent on participants’ allocation decisions but was preprogrammed. When participants made altruistic decisions in the donation task, they received positive feedback, and when they made selfish decisions, they received negative feedback. We were only interested in negative feedback trials in the current research.

Data, code, materials, and preregistration can be found at https://osf.io/9kmgd/?view_only=f35e1fb454f2471ea8c7432388e38e57.

#### Participants

**Fig. 1:**
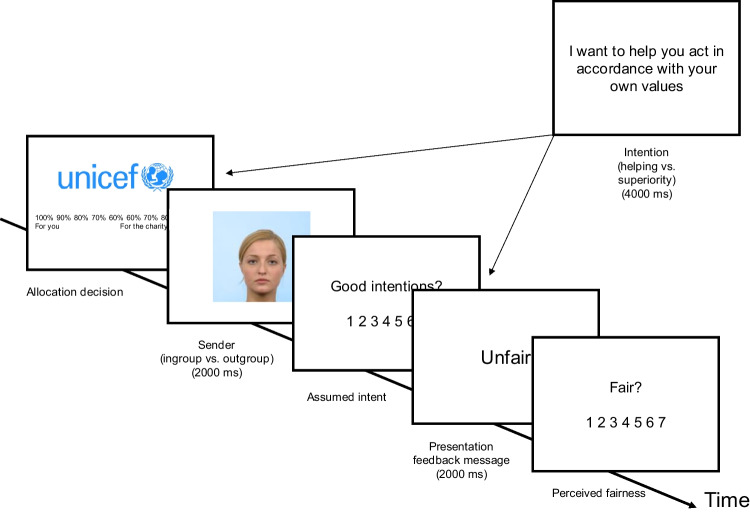
Example trial Study 1

Dutch participants took part in the study on the platform Prolific (*N* = 50) in exchange for €5 (for sensitivity power simulations, see SI). Six participants were excluded, because they did not meet the inclusion requirement for our experimental paradigm to find it important to give money to people in need. The remaining sample consisted of 44 participants (*M*_age_ = 26.55, *SD* = 8.17, 17 females, 1 other): 13 participants were in the condition where intentions were presented directly before the sender was presented; 16 participants in the condition where intentions were presented directly before the feedback message; and 15 participants in the control condition where no intentions were presented (Fig. 1).

#### Procedure, instruments, and stimuli

The current research tested whether communicating intentions can decrease recipients’ defensive responses toward negative feedback on their morality when feedback is delivered by an outgroup member. We adapted an experimental paradigm from previous research (Rösler et al., 2023; Schindler & Kissler, 2018). As in previous research (Rösler et al., 2023), we explained to participants that the senders and feedback messages originated from a previous experiment. In this previous experiment, participants were presented with behavioral decisions of targets and were asked to judge and provide feedback on the character of these targets. In the current experiment, participants would receive the judgments that were given to targets who made similar decisions as they did in the previous experiment. We emphasized that the feedback messages therefore would be representative of how feedback senders from the previous experiment would judge their character. We further explained, for the two conditions where intentions were communicated, that these previous participants were asked to select predescribed intentions of why they gave this feedback and that these intentions were presented in the current task as well.

The experiment was presented on Gorilla, lasted 30 minutes, and was approved by the local Ethics Review Board. Participants gave informed consent and were fully debriefed and thanked. The main task consisted of 80 trials, 20 trials per condition of the two within-participants factors of our experimental design (i.e., 2: Sender’s group-membership x 2: Communicated intentions). Because we were interested in negative feedback in the current research, we excluded positive trials from further analyses. Because the valence of the feedback depended on participants’ allocation decisions in the task, the actual trial number included in the analysis slightly varied per participant. On average, this left us with 13 trials for the ingroup helping intention condition, 13 trials for the outgroup helping intention condition, 13 trials for the ingroup moral superiority intention condition, and 12 trials for the outgroup moral superiority intention condition. For considerations on reliability of measures, see SI.

At the beginning of the experiment, we asked participants about their prosocial orientation (“I find it important to give some money to people in need”; 7-point Likert scale, 1 = fully disagree, 7 = fully agree). A score > 4 on this measure was a prerequisite for taking part in the experiment to ensure the credibility of our intention manipulation. Participants then completed the main task.

Each trial started with the presentation of a charity and participants were asked to allocate a hypothetical amount of money to themselves and to the charity (Fig. 1). This was followed by a picture of a feedback sender’s face with the background color indicating ingroup or outgroup membership (2000 ms). Then, the variable *assumed good intentions* was measured. After that, the character trait indicating the moral feedback was shown (2000 ms). Finally, we measured how *fair participants perceived the feedback* to be. Depending on the condition, a sentence displaying the sender’s intention was presented in the trial at different time points (i.e., before the sender was presented or before the feedback was presented, for 4000 ms), or no intention was presented.

##### Allocation decisions

At the beginning of each trial, participants were asked to divide a hypothetical amount of €20 between themselves and a charity.^2^ We listed 80 Dutch charitable organizations related to causes such as a green environment, medical research, or animal rights. We presented charity names and logos together with a short explanation of the charitable cause. Participants could choose percentage amounts presented on a 10-point scale with a 10-point distance between percentages (e.g., 10%, 20%, 30%). Each choice implied both the amount for the self and the charity. To demonstrate, if a participant would choose to allocate 80% (i.e., €16) to the charity, 20% (i.e., €4) would go to their experiment account. We did not give participants the option to equally divide the money between themselves and the charity. To ensure variability in participants’ allocation decisions and because we were only interested in selfish decisions, we added an €0.57 (0.50 GBP) incentive for the 10 participants who would contribute the least—consistent with keeping more money to themselves and modeling real-life considerations of donating to charities.

##### Sender’s group-membership

To manipulate the senders’ group-membership, we measured participants’ social value orientation (SVO; Messick & McClintock, 1968; Van Lange, 1999) with the SVO slider measure (Murphy et al., 2011). We then explained that the senders of the feedback messages would either belong to the ingroup, meaning the sender has a similar SVO-type as the participant or to the outgroup, meaning the sender has a different SVO-type than the participant (see SI for participants’ SVO types). Participants did not receive feedback on their own SVO-type, and we did not specify the SVO-type of outgroup members.

To check whether participants indeed identified with these groups, we measured identification with people having a similar/different SVO-type with three items (e.g., “I identify with other members of this group”; Ellemers, Kortekaas, & Ouwerkerk, 1999, ingroup, α = .74, outgroup, α = .74), directly after introducing the group memberships to participants.

To represent ingroup and outgroup members in the experimental task, we selected 20 Caucasian faced-forward faces (10 females, 10 males) from the Radboud face database (Langner et al., 2010). We changed the background color of half of the faces to blue to indicate ingroup membership of the faces and the other half to yellow to indicate outgroup membership of the faces. Attractiveness of faces did not vary between gender and assigned social group-membership of faces, *Fs* ≤ 2.22, *ps* ≥ .156.

To learn these group-memberships, participants were asked to perform a social categorization task in which they had to correctly categorize each sender’s face as belonging to the ingroup or outgroup twice.

##### Feedback

For the feedback, we used 32 Dutch moral traits, 16 positive traits, and 16 negative traits, from previous research (see SI). The traits had been pretested on and did not vary between their familiarity, length, frequency (per million), and the level of arousal associated with them (Rösler et al., 2023). Trials, in which participants allocated most of the money to the charity (>70% of the €20), were coded as altruistic. Participants then received a positive moral feedback message (e.g., cooperative). When participants did not allocate most of the money to the charity (<70% of the €20), these decisions were coded as selfish and participants received a negative moral feedback message (e.g., uncooperative).^3^

##### Communicated intentions

The focus of our research was on negative feedback. However, the valence of the feedback depended on participants’ allocation decisions and participants received both negative and positive feedback messages to increase the credibility of the feedback. The displayed intentions of senders therefore also pertained to both the positive and negative feedback messages. That is, for positive feedback after altruistic decisions, intentions were related to acknowledging that the participant had contributed most of the money to the charity (e.g., “It seems like it is important for you to donate to charities”). More importantly, for negative feedback after selfish decisions, these intentions were related to either helping intentions (e.g., “I want to help you by reminding you that you said it is important for you to donate to charities”) or moral superiority intentions (e.g., “I seem to be a better person than you are”). We pretested intention sentences for their representativeness for feedback senders’ intentions among a Dutch sample (*N* = 20, *M*_age_ = 31.55 years, *SD* = 9.52, 8 females, on Prolific) and selected the final set based on this pretest (Table 1). We measured participants’ assumptions about the sender’s intentions with one item (“Do you think this person had good intentions when (s)he evaluated you based on your decision?”; 7-point scale, 1 = not at all, 7 = very much).

**Table 1:** Means, standard deviations, and F-statistics for sentence stimuli per intention condition

To what extent is the sentence representative of each of the following intentions? (1 = not at all, 7 = very much)	Helping sentences (*N* = 5)	Superiority sentences (*N* = 5)	Acknowledgment sentences (*N* = 10)	*F*(2, 17)
Helping you improve	5.15 (.19)	2.01 (.25)	4.30 (.26)	225.77***
Showing moral superiority	3.98 (.19)	6.64 (.10)	2.22 (.32)	493.22***
Acknowledging that you gave money	2.98 (.12)	2.71 (1.02)	5.73 (.54)	52.14***

Each sentence was rated by 20 participants for whether it was representative of a helping, moral superiority, or acknowledgment intention (the latter not relevant for the current research as they were shown after positive feedback) with a 7-point Likert scale (1 = not at all, 7 = very much). ***p < .001

##### Perceived fairness of negative moral feedback

After the feedback message was shown, we measured the perceived fairness of this message with one item (“Do you think this evaluation of your character, based on your decision, was fair?”; 7-point scale, 1 = not at all, 7 = very much). Please note that we hence interpret scores below the midpoint of the scale (4) as unfair.

##### Data analyses

To test our hypotheses, we fitted linear mixed models (LMM) to our data with maximum-likelihood estimation. We used the lme function of the nlme package (Pinheiro et al., 2023) in R (version 4.0.0). Model assumptions were checked using the model_check function of the performance package (Lüdecke et al., 2023). We accounted for repeated measures by adding the participant number as a random intercept to all models. For all analyses, we selected trials in which people made selfish (rather than altruistic) choices and therefore received negative feedback. LMMs were computed on single trial data. We used Mahalanobis distance to check for outliers and detected none.

### Results

#### Checks

##### Ingroup/outgroup identification

As intended, a paired-sample *t*-test revealed that participants identified more with ingroup senders (*M* = 4.63, *SD* = 1.88) than with outgroup senders (*M* = 2.85, *SD* = .93), *t*(43) = 9.65, *p* < .001, 95% CI = [1.41, 2.15].

##### Communicated intentions

We checked whether our intention manipulation was successful by selecting data from the condition where participants were presented with the intentions of senders before being asked to judge their good intent. We then checked whether participants assumed better intent in trials where senders communicated the intention to help the participant, rather than their moral superiority. That was indeed the case: an LMM specifying communicated intentions (helping vs. superiority intention) as a fixed effect and assumed intentions as a dependent variable revealed that participants assumed better intentions of senders who had communicated their intention to help than their moral superiority, *B* = −1.87, *t* = −16.85, *p* < .001, 95% CI = [−2.09, −1.65].

#### Assumed intent and perceived fairness

##### Assumptions about intentions of senders

We predicted that participants would assume worse intentions of outgroup senders than ingroup senders when senders did not communicate their intentions. However, we predicted that this effect would be attenuated when senders would make their intentions explicit. Because the factor “type of communicated intention” was manipulated only within two of the three between-participants conditions, we examined this prediction with a two-step approach.^4^

**Fig. 2:**
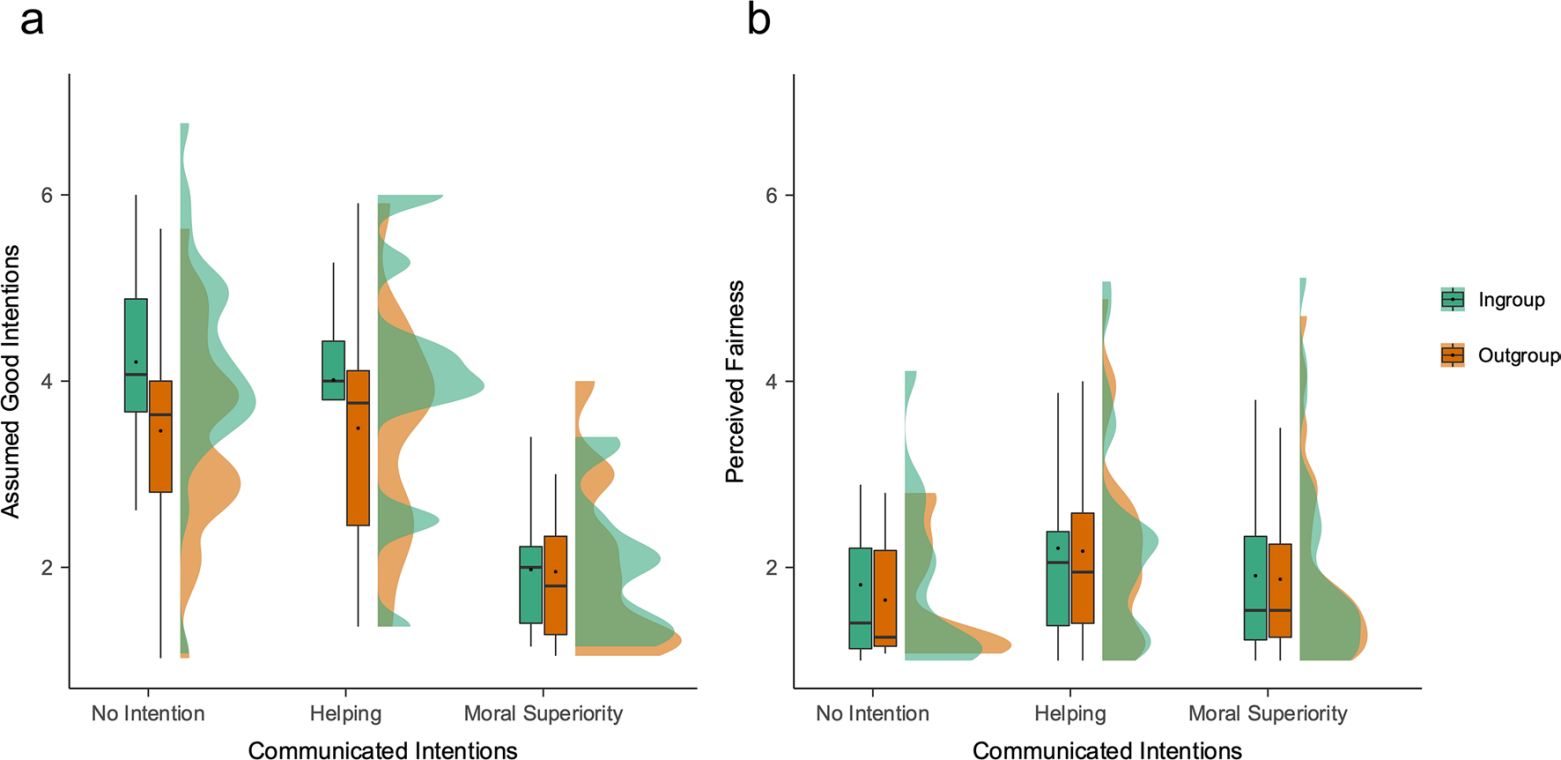
Raincloud plots for the effects of feedback sender’s group membership (i.e., ingroup vs. outgroup) and their communicated intentions (i.e., helping vs. moral superiority intention vs. no intention) on assumed good intentions and on perceived fairness of negative moral feedback. *Note.* We display a boxplot, mean scores, and half-density distributions. Please note that the “No Intention” condition was manipulated between-participants and the “Helping” and “Moral Superiority” condition within-participants. As predicted, (**a**) when senders did not communicate their intentions, participants assumed worse intentions from outgroup senders than ingroup senders and (**b**) perceived their feedback as significantly more unfair. When intentions were explicitly communicated, the group-membership effect was not observed for either (**a**) assumed intent or (**b**) perceived fairness. When senders expressed a willingness to help rather than conveyed a sense of moral superiority, (**a**) participants tended to infer more positive intentions from senders and (**b**) they reported reduced perceptions of unfairness regarding negative feedback concerning their morality

First, we selected data in which participants *were not, or not yet,* presented with the sender’s intentions in the task. We fitted LMMs predicting assumed intentions specifying the sender’s group membership (ingroup vs. outgroup) as a fixed effect. As expected, participants assumed that outgroup senders had worse intentions than ingroup senders before receiving feedback from them, *B* = −.74, *t* = −11.38, *p* < .001, 95% CI = [−.87, −.62] (Fig. 2a).

We then selected data from the condition where participants were presented with intentions before they rated assumed intent, computing the same model. In line with our predictions, the effect of group-membership was not significant, *B* = −.13, *t* = -0.95, *p* = .344, 95% CI = [−.40, .14],^5^ when intentions were made explicit, and linear hypothesis testing revealed that this estimate was significantly reduced compared to the effect of sender’s group membership in the control condition, χ^2^ (1) = 20.43, *p* < .001. Moreover, participants assumed better intentions of senders who had communicated their intention to help than their moral superiority, *B* = −1.87, *t* = −16.85, *p* < .001, 95% CI = [−2.09, −1.65]. These findings suggest that when senders express their intentions while providing negative feedback regarding someone else’s morality, this can reduce the impact of their group membership on the assumptions formed by the recipient about the sender’s intentions. Moreover, they suggest that expectations of senders’ intentions are more positive when senders express wanting to help compared with their superiority.

##### Perceived fairness of negative moral feedback messages

We used the same two-step approach to investigate whether participants perceived negative feedback on their selfish decisions as more unfair when outgroup senders than ingroup senders delivered feedback and whether such an effect would be attenuated when senders made their intentions explicit.^6^ Then, we checked whether participants perceived this negative feedback as less unfair when senders communicated their intention to help, rather than conveyed their moral superiority.

We first selected data from the control condition, where no intentions were communicated, and computed the same model as above. As expected, participants perceived negative feedback messages as more unfair when these were delivered by outgroup senders than by ingroup senders, *B* = −.16,* t* = −2.43, *p* = .015, 95% confidence interval (CI) = [−.30, −.03] (Fig. 2b).

We then selected data from the two between conditions where participants were presented with intentions in the task, computing the same model as for assumed intent, but adding the factor time of communicating intentions (before sender was presented vs. before message was presented, between-participants) as a fixed effect. In line with our predictions, there was no effect of sender’s group membership on how unfair participants perceived negative feedback messages, *B* = −0.03,* t* = −.45, *p* = .656, 95% CI = [.70, 1.19].^7^ However, statistically comparing the size of the estimate to the estimate of the effect of sender’s group membership in the control condition did not reveal any significant differences, χ^2^ (1) = 1.92, *p* = .166. There also was no interaction between group membership and the type of intention, *B* = −0.08,* t* = −.43, *p* = .669, 95% CI = [−.46, .29], and linear hypothesis testing showed that there was no significant difference between ingroup (*M* = 2.33, SD = 1.87) and outgroup senders (*M* = 2.22, SD = 1.77), *z* = −1.59,* p* = .112. As predicted, in the condition where intentions were made explicit, participants perceived negative feedback as less unfair when senders communicated their intention to help rather than their moral superiority, *B* = −.25, *t* = −3.99,* p* < .001, 95% CI = [−.37, −.12]. The timepoint when the intention was communicated in the trial did not influence any of our variables and is not further discussed.

These findings potentially indicate a reduction in the impact of the sender’s group membership on the evaluations of their feedback fairness, mirroring the reduced impact observed in the expectations of feedback senders’ intentions. However, one must be cautious in the interpretation of this finding, because linear hypothesis testing showed that the estimates were not significantly different from each other. Moreover, the findings indicate that when senders express an intention to help, their feedback is perceived as less unfair in contrast to when they express superiority. Finally, the nonsignificant interaction between sender’s group membership and the type of intention and the means of perceived fairness of feedback from both ingroup and outgroup senders being of equal size may suggest that expressing intentions to help is equally effective for both ingroup and outgroup senders in enhancing perceived fairness.

### Discussion

The results of Study 1 demonstrate that when outgroup members communicate their intention to help when criticizing others on their morality, this enhances the receipt of such feedback. That is, participants were less receptive to feedback from outgroup compared with ingroup senders when these did not make their intentions explicit. However, when feedback senders’ intentions were made explicit, the effect of the sender’s group membership was nonsignificant for both assumed intentions of feedback senders and perceived fairness of their feedback, as well as significantly reduced for assumed intentions compared to the condition where no intentions were made explicit.

Moreover, negative moral feedback was perceived as less unfair when (both ingroup and outgroup) senders communicated the intention to help the participant rather than their own moral superiority (for exploratory analyses on whether communicated intentions also influenced behavioral donation choices, see SI). Together, these observations corroborate our main predictions and imply that explicitly stating one's intentions can reduce the influence of the sender’s group membership on how their negative feedback is perceived. Specifically, as predicted, once explicit intentions are communicated, senders who state helping intentions rather than moral superiority intentions, can enhance recipients’ perception that negative feedback pertaining to their morality might be fair.

However, only investigating self-report answers might not reveal how participants truly experienced the feedback (Ellemers & van Nunspeet, 2020) and cannot give insight into the underlying cognitive mechanisms that may lead to decreased defensiveness in recipients. To test this, in the second study, we investigated participants’ continuous brain activity while they received feedback that was delivered with different types of intentions by recording an electroencephalography (EEG). Another drawback of Study 1 relates to how the control condition was implemented in the design. Because we aimed to have a group of participants taking the task without being presented with any intentions, the control condition was part of a between-participants factor. However, this implies that in our analysis we compared communicating helping intentions to communicating moral superiority intentions and that our findings could be explained by a contrast effect as we cannot directly compare either to the control condition. Lower scores of perceived unfairness for feedback where helping intentions were communicated could potentially stem from the comparison group (i.e., superiority intentions) being perceived as very negative. In Study 2, we addressed this issue by including the control condition in the within-participants factor of “communicated intentions.”

## Study 2

Study 2 used the same donation task as Study 1. We extracted event-related potentials (ERPs) from the EEG to examine the cognitive processes associated with receiving feedback. This enabled us to test whether the type of intention communicated by the feedback senders modulated the subsequent cognitive processing of negative moral feedback messages. Because the feedback in our study was presented as judgments of participants’ character in the form of trait words, we decided to focus on ERPs elicited by viewing emotional words.

### Event-related potentials

Higher cognitive functions in response to viewing emotional words are associated with the P300 and the LPP. That is, the P300 is associated with selective attention and working memory updating (Lang et al., 1997; Polich, 2007), and the LPP is associated with sustained attention and decoding of affective meaning (Schupp et al., 2004; Schupp, Flaisch, Stockburger, & Junghöfer, 2006). According to the framework of “motivated attention,” these two ERPs are indicative of how much arousal is evoked by a stimulus and how deeply people process the information (Lang, Bradley, & Cuthbert, 1997; Lang & Bradley, 2013). These assumptions are based on the finding that people show increased P300 and LPP amplitudes to highly arousing positive and negative compared with less arousing or neutral stimuli (Lang et al., 1997; Lang & Bradley, 2013; Schupp et al., 2004).

### Feedback processing

The words in our study represented feedback messages. Research on feedback processing has examined how ERPs can give insight into the attentional processing of feedback messages delivered by senders with different characteristics (Schindler et al., 2020; Schindler & Kissler, 2018). Participants in this previous work attended more to feedback messages delivered by a more self-relevant sender (i.e., a human) than a less relevant sender (i.e., a computer), which was indicated by larger P200, P300, and LPP amplitudes in response to viewing feedback messages. Thus, characteristics of the social context, such as the self-relevance of a feedback sender can influence participants’ attentional processing of the feedback they receive.

Building on this work, we tested whether communicating feedback senders’ intentions alongside the feedback can modulate the processing of such feedback messages in a similar vein. We predicted that when senders would communicate their intention to help rather than their moral superiority or no intention, this would increase participants' attentional processing of the feedback messages. Specifically, we predicted this type of feedback, provided in a social context, to be perceived as more self-relevant and hence to elicit greater P300 and LPP amplitudes.^8^

Additionally, although we had not preregistered hypotheses about initial attentional processing, we explored the modulation of the P200 as this was apparent during visual inspection of the ERP waveforms. P200 amplitudes have been associated with the initial lexical encoding of words (Kissler, Assadollahi, & Herbert, 2006; Trauer, Andersen, Kotz, & Müller, 2012). For example, in an experiment in which participants were presented with task-irrelevant words, negative (vs. neutral) words elicited enhanced P200 amplitudes, suggesting that the valence and “emotionality” of negative words can facilitate the semantic encoding of it (Trauer et al., 2012). In research on the social categorization of faces, larger P200 amplitudes elicited by viewing racial outgroup (vs. ingroup) faces have been associated with “rapidly occurring vigilance,” because the P200 indicates attention deployment (Ito & Bartholow, 2009). Because feedback messages delivered in a helpful context may be perceived as less threatening than feedback messages delivered in a harmful context, they may decrease such attentional vigilance as indicated by decreased P200 amplitudes.

### Method

#### Design and participants

The experiment had a 2: Feedback sender’s group-membership (in- vs. outgroup) x 3: Communicated intentions (helping vs. superiority intentions vs. control [no intention communicated]) factorial design. In this study, all factors were manipulated within participants to be able to compare helping and moral superiority intentions to a baseline score, namely the control condition where no intentions were communicated. Because the same senders sometimes communicated their intentions and sometimes not, this means that testing for the effect of sender’s group membership in the control condition is more difficult than in Study 1 and represents a slightly different comparison. As in Study 1, ingroup senders were specified as having a similar SVO-type, and outgroup senders as having a different SVO-type. The primary emphasis of this study was on investigating the impact of manipulating intentions, because Study 1 revealed that when intentions are explicitly stated, evaluations of feedback are less influenced by the sender’s group membership. The trials were presented in a before-the-experiment randomized order. This order was the same for all participants.

A total of 38 right-handed Dutch participants with no history of neurological or psychiatric problems and normal-to-corrected vision took part in the experiment in exchange for €18 or student credit.^9^ As in Study 1, it was necessary for the credibility of our intention manipulation that participants found it important to give money to people in need. One participant indicated that they did not find it important (i.e., a score < 4 on the scale of our measure) and was therefore excluded from further analyses. Three participants had to be excluded because of technical problems. Therefore, the behavioral sample consisted of 34 participants (*M*_age_ = 23.48, *SD* = 4.17, 27 female, one participant not indicating age). For the behavioral data, we ended up with, on average, 47 trials for the control condition, 28 for the moral superiority intentions condition, and 36 trials for the helping intentions condition.

For the EEG analyses, we had to exclude 13 more participants as the data retained too few observations to achieve a good signal-to-noise ratio after preprocessing (i.e., <30 trials per condition as specified in preregistration; Luck, 2005). Thus, we ended up with an EEG sample of 21 participants. This high attrition was the result of the unbalanced design, as trials included in the analyses depended on participants’ choices in the paradigm (i.e., to be able to focus on participants’ processing of negative feedback, we only included trials in which they made selfish decisions). Each condition therefore had at least 30 trials (because we had to exclude so many participants, we made this criterion a bit more lenient and included a couple of participants who had 28 and 29 trials left in one of the conditions). The study was approved by the local ethics review board. Participants were fully debriefed and thanked for their participation.

#### Procedure, instruments, and stimuli

The procedure was very similar to Study 1. We made a few adjustments to the stimuli, dependent variables, and the length of the experiment to measure an EEG: We increased the trial number to 170 to be able to examine ERP responses. For that reason, we also extended the list of charities for which participants were asked to make an allocation decision. We matched the feedback sender’s gender to the participant’s gender. We counterbalanced the faces displayed as ingroup members and outgroup members. That is, the same set of faces was shown to half of the participants as belonging to the ingroup, whereas for the other half of the participants, it was shown as belonging to the outgroup. Additionally, we included an affirmation phase in each trial (Rösler et al., 2023; Schindler & Kissler, 2018). We explained to participants that the feedback senders were presented with several traits and had to select which traits they thought were representative of the character of the participant—based on the participant’s allocation decision. We used this affirmation phase to ensure that ERPs were related solely to receiving the actual feedback, rather than the sensory language processing of the trait. In this study, we incentivized selfish decisions by paying an additional €1 to participants who contributed less than 30% of all contributions possible. Finally, we did not measure “assumed intentions” in this study.

**Fig. 3:**
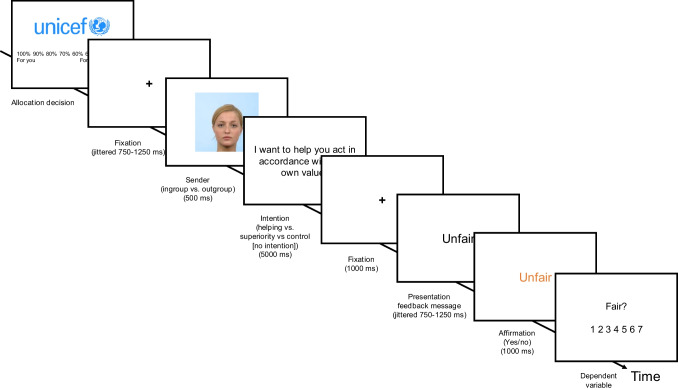
Example trial Study 2

Each trial started with the presentation of the charity, and participants were asked to allocate a hypothetical amount of money between themselves and the charity (Fig. 3). This was followed by a fixation cross (jittered duration, 750–1250 ms), a picture of a sender’s face with the background color indicating ingroup or outgroup membership (500 ms), an intention (or no intention, in the control condition, 5000 ms), followed by a fixation cross (1000 ms). Then, the feedback message (i.e., in form of a character trait) was first presented to the participant (jittered duration, 750–1250 ms) and then either affirmed (i.e., turned orange) or not affirmed (i.e., stayed black, 1000 ms). As explained above, this affirmation phase ensured measuring responses to the actual feedback message. Finally, *perceived fairness of feedback* was measured as dependent variable.

#### EEG acquisition

We recorded EEG from 35 electrodes embedded in a stretch head cap and positioned according to the 10-10 system: F7, F3, F1, Fz, F2, F4, F8, FC3, FC1, FCz, FC2, FC4, T7, C3, C1, Cz, C2, C4, T8, CP3, CP1, CPz, CP2, CP4, P7, P5, P3, P1, Pz, P2, P4, P6, P8, POZ, and Oz. For the recording of the signal, we used the Biosemi active-electrode system with a sampling rate of 256 Hz. This system uses an analog hardware filter at one third of the sampling frequency to prevent aliasing. For ground recording voltages and initial referencing, we used CMS and DLR. Electrode impedance was kept below 5 kΩ. We recorded horizontal and vertical eye movements to correct for eye movement. EEG activity was recorded with ActiView software.

Offline, we analyzed the EEG using Brain Vision Analyzer 2.1 (Brainproduct GmbH, Munich, Germany). The EEG was re-referenced to the average of the left and right mastoids. We used the regression approach (Gratton et al., 1983) to correct for ocular artifacts, filtered the signal (0.01–30 Hz), and rejected trials with movement artifacts. We created separate stimulus-locked epochs for the feedback stimulus −200 ms before 800 ms after the event. Epochs were averaged and baseline corrected with the averaged signal 200 to 0 ms before the event. Lastly, we created separate epochs for each of the three relevant conditions (i.e., helping vs. superiority intentions vs. control [i.e. no intentions]) of our experimental paradigm.

#### EEG analyses

Our experimental design was unbalanced, because the exact trial number per condition was dependent on participants’ allocation decisions. This led to a different number of trials between conditions; therefore, we used mean rather than peak amplitudes for the ERP analyses to avoid bias in noise levels (Luck & Kappenman, 2018).

ERPs were time-locked to when the trait word switched from black to orange, indicating that the trait had been affirmed by the feedback sender as pertaining to the participant’s character. Trials with nonaffirmed feedback messages were excluded from further analyses. P200 amplitudes were largest at frontocentral and centroparietal electrodes Fz, FCz, Cz, and CPz (consistent with past literature; Herbert et al., 2006; León et al., 2010; Rösler et al., 2023). Mean amplitudes for the P200 were averaged between 200–230-ms poststimulus onset. Consistent with past literature (Polich, 2007; Rösler et al., 2023; Schupp et al., 2004, 2006), P300 and LPP amplitudes were largest at centroparietal electrodes Pz, CPz, and Cz. Mean amplitudes for the P300 were averaged between 300–400 ms and for the LPP between 400–700-ms poststimulus onset.

LMMs for EEG data were computed on aggregated trial data (i.e., a single mean score per condition and electrode per participant) and LMMs for behavioral data on single trial data.

### Results

#### Checks

As intended, a paired sample *t*-test showed that participants identified more with ingroup senders (*M* = 4.65, *SD* = 1.02) than with outgroup senders (*M* = 2.52, *SD* = .93), *t*(33) = 10.77, *p* < .001, 95% CI = [1.73, 2.53].

#### Perceived fairness of negative moral feedback messages

We first tested whether we could replicate that participants perceive negative feedback on their selfish decisions as less unfair when senders stated their intention to help (vs. their moral superiority or not stating intentions). We computed the same model as in Study 1 excluding the timepoint of stated intentions and the factor communicated intentions included three levels (helping vs. superiority intention vs. no intention).

**Fig. 4:**
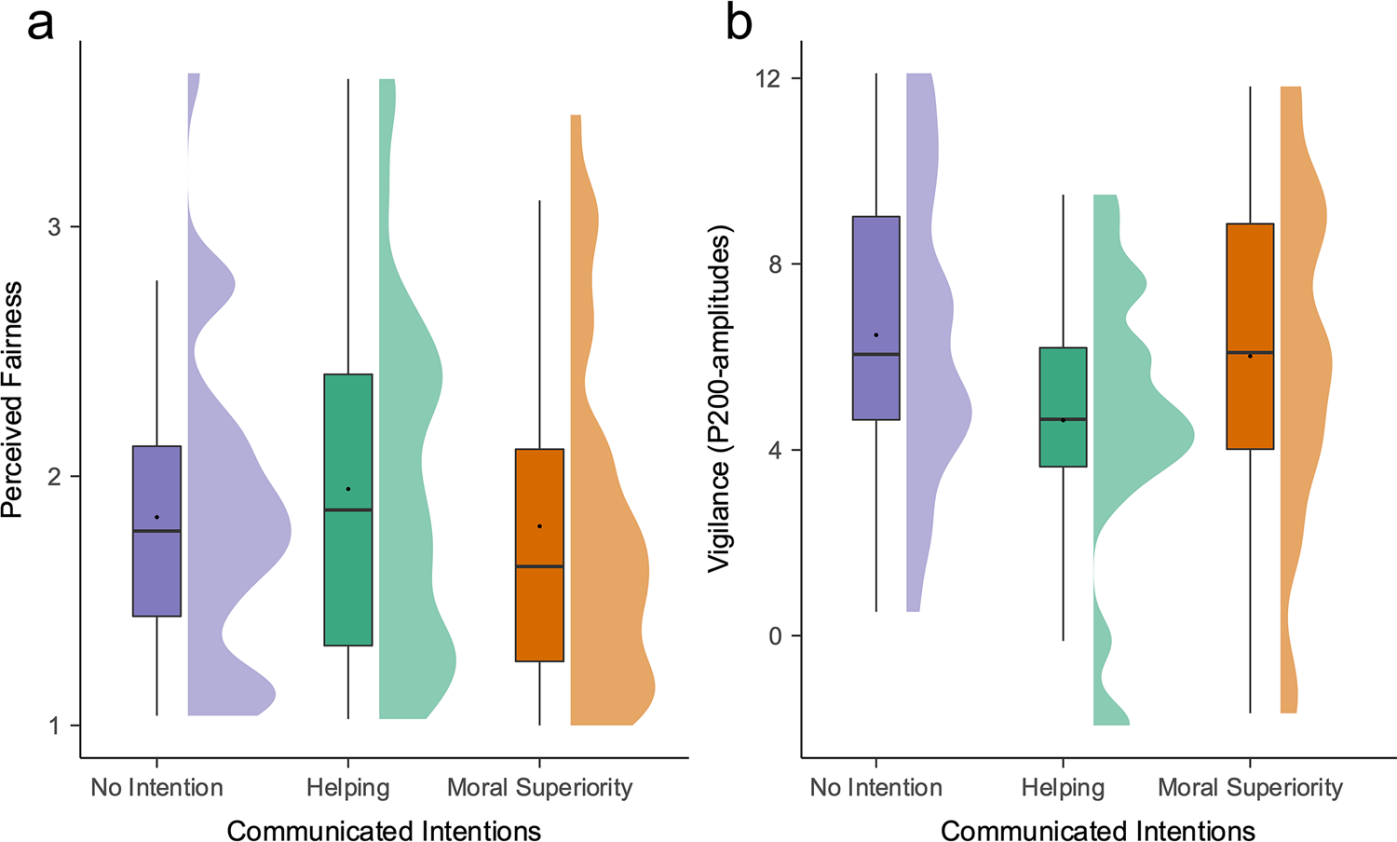
Raincloud plots for the effects of communicated intentions (i.e., helping vs. moral superiority intention vs. no intention) on (**a**) perceived fairness of and (**b**) vigilance (as indicated by P200 amplitudes) in response to negative feedback on participants’ selfish decisions. *Note.* Boxplot, mean scores, and half-density distributions. In trials in which senders had communicated their intention to help, participants perceived negative moral feedback as less unfair and were less attentionally vigilant when semantically processing the feedback messages compared with trials in which senders did not communicate their intentions or communicated their moral superiority

Replicating Study 1, participants perceived negative feedback messages as less unfair when senders communicated helping intentions compared to not communicating any intentions, *B* = .09, *t* = 2.34, *p* = .019, 95% CI = [.01, .16], and compared with communicating moral superiority intentions, *B* = .10, *t* = 2.19, *p* = .029, 95% CI = [.01, 1.78] (Fig. 4). There was no significant difference between not communicating intentions and communicating moral superiority intentions, *B* = −0.005, *t* = −0.12, *p* = .901, 95% CI = [−.09, .075]. As in Study 1 in the conditions where intentions were made explicit, the sender’s group membership did not predict perceived fairness, *B* = .09, *t* = 1.86, *p* = .063, 95% CI = [−.005, .19], and there was no interaction with the type of intentions delivered, Group-membership x Intentions (helping vs. control):* B* = −.04, *t* = −.54, *p* = .588, 95% CI = [−.19, .11], Group-membership x Intentions (superiority vs. control): *B* = −.05, *t* = .56, *p* = .577, 95% CI = [−.21, 1.12]. Linear hypothesis testing showed that there was no significant difference between ingroup (*M* = 1.80, *SD* = 1.10) and outgroup senders (*M* = 1.87, *SD* = 1.24), *z* = 1.94,* p* = .052. These results are in line with Study 1, because they suggest that stating one’s intention to help proves to be a successful strategy for both ingroup and outgroup senders in enhancing the perceived fairness of their feedback.

### Event-related brain potentials

#### Exploratory analysis: P200

**Fig. 5:**
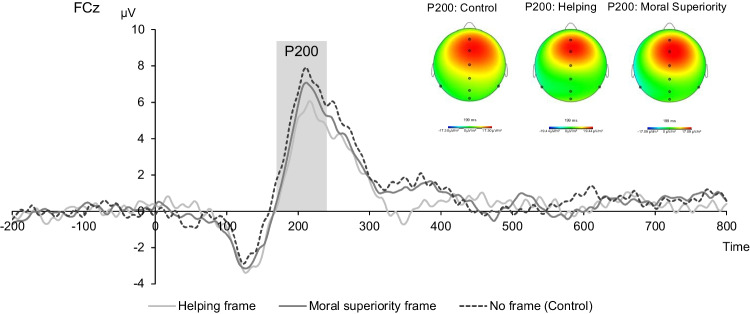
ERP waveforms and topographical maps for negative moral feedback messages at frontal midline electrode FCz for helping, superiority, and no intentions communicated (i.e., control condition)

Visual inspection of the data in the time window 200–230-ms poststimulus-onset showed potentially interesting changes in the P200 amplitudes for the factor “communicated intentions.” We examined whether P200 amplitudes were modulated by this factor using an LMM to predict mean amplitudes. We submitted communicated intentions (helping vs. superiority intention vs. control [no intention communicated]) and electrode site (Fz vs. FCz vs. Cz vs. CPz) as fixed effects to this model. Indeed, communicated intentions modulated P200 amplitudes elicited by the negative moral feedback messages (see Figs. 4 and 5, see SI for standard deviations around averages). Compared with control trials, negative moral feedback messages elicited lower P200 amplitudes in trials in which senders communicated their intention to help, *B* = −1.84, *t* = −5.89, *p* < .001, 95% CI = [−2.45, −1.23]. Similarly, communicating helping intentions also decreased P200 amplitudes compared with communicating moral superiority, *B* = −1.38, *t* = −4.42, *p* < .001, 95% CI = [−1.99, −.77]. Amplitudes did not significantly differ when superiority or no intentions were communicated, *B* = .46, *t* = 1.46, *p* = .145, 95% CI = [−1.15, 1.06]. There were main effects of electrode site. The electrodes FCz, Cz, and Fz elicited larger amplitudes (*B* = 2.17, *t* = 6.02, *p* < .001, 95% CI = [1.47, 2.87], *B* = 1.62, *t* = 4.49, *p* < .001, 95% CI = [.92, 2.32], *B* = 1.24, *t* = 3.45, *p* < .001, 95% CI = [.54, 1.95], respectively) than CPz, all other *Bs* < .49, *ps* > .581.

#### P300 and LPP

**Fig. 6:**
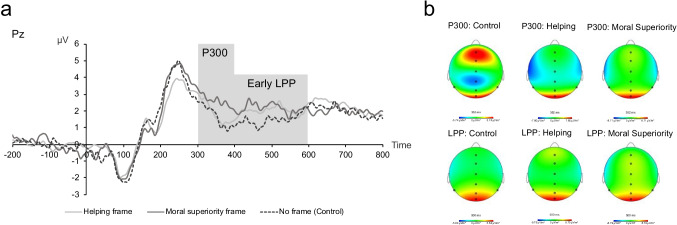
ERP waveforms and topographical maps for negative moral feedback messages at parietal midline electrode Pz for helping, superiority, and no intentions communicated (i.e., control condition)

We submitted communicated intentions (helping vs. superiority intention vs. control [no intention communicated]) and electrode site (Pz vs. CPz vs. Cz) as fixed effects to a model predicting P300 mean amplitudes (for results on P3a, see SI). Contrary to our predictions, communicating helping (vs. no) intentions did not increase P300 amplitudes, *B* = −.28, *t* = −1.01, *p* = .315, 95% CI = [−.83, .26]. Rather, when senders communicated their moral superiority, this increased P300 amplitudes compared with the control condition, *B* = .82, *t* = 2.93, *p* = .004, 95% CI = [.28, 1.37], and compared with when they communicated the intention to help, *B* = −1.10, *t* = −3.94, *p* < .001, 95% CI = [−1.65, −.56] (see Fig. 6, see SI for standard deviations around averages and density plots). There also was a main effect of electrode: compared with CPz, Pz amplitudes were larger, *B* = .75, *t* = 2.70, *p* = .007, 95% CI = [.21, 1.30], all other *Bs* < .46, *ps* > .505.

We also used an LMM to predict mean LPP amplitudes. We submitted communicated intentions (helping vs. superiority intention vs. no intention) and electrode site (Pz vs. CPz vs. Cz) as fixed effects to this model. There were no effects of communicated intentions (helping vs. no intentions: *B* = .27, *t* = 1.99, *p* = .232, 95% CI = [−.17, .70], moral superiority vs. no intentions: *B* = .39, *t* = 1.76, *p* = .080, 95% CI = [−.04, .83]. There was a main effect of electrode: compared to CPz, Pz amplitudes were larger, *B* = .56, *t* = 2.51, *p* = .013, 95% CI = [.13, 1.00], and Cz amplitudes were smaller, *B* = −.70, *t* = −3.14, *p* = .002, 95% CI = [−1.14, −.27]. Visually inspecting the grand averages (Fig. 6) seemed to suggest a similar effect as for the P300, with higher LPP amplitudes elicited when senders communicated moral superiority intentions (vs. helping and no intentions) for the earlier LPP (400–600 ms) rather than the later LPP (600–800 ms). A separate analysis for the early LPP revealed that compared with the control condition, communicating moral superiority intentions increased early LPP amplitudes, *B* = .69, *t* = 2.60, *p* = .010, 95% CI = [.17, 1.20]. There were no significant differences between the helping and superiority intentions condition, *B* = .35, *t* = 1.32, *p* = .188, 95% CI = [−1.68, .87], and helping intentions and the control condition, *B* = .34, *t* = 1.28, *p* = .203, 95% CI = [−1.18, .85].

The P300 and LPP findings indicate an unexpected trend: while we anticipated increased motivated attention to feedback provided with the intention to help, participants exhibited heightened motivated attention toward negative feedback when the senders conveyed their moral superiority.

### Discussion

In Study 2, we replicated the finding of Study 1 that participants perceived negative feedback on their morality as less unfair when senders communicated their intention to help rather than their moral superiority. Moreover, we demonstrated that this effect is not only caused by a mere contrast effect that occurs because we compared helping intentions to moral superiority intentions—the latter potentially inducing an extremely negative emotional response. Participants also perceived negative feedback that was accompanied by senders’ helpful intentions as less unfair when directly compared with negative feedback that was not accompanied by senders’ intentions. As in Study 1, reported perceived fairness was very similar for both ingroup and outgroup senders, suggesting that communicating helping intentions works well to increase perceived fairness of negative moral feedback, regardless of whether the sender belongs to the ingroup or outgroup.

Complementing these self-report findings, the exploratory P200 findings may suggest that negative moral feedback messages were semantically processed with less vigilance when senders communicated their intention to help compared with when senders did not communicate their intentions or communicated their moral superiority. The P300/LPP findings did not show enhanced motivation to attend to feedback messages that were provided with the intention to help, as initially expected, but enhanced motivation to attend to messages delivered with moral superiority.

Taken together, the results from Study 2 confirm those of Study 1, showing that participants perceived negative moral feedback as less unfair when feedback senders explicitly communicated their intention to help than their moral superiority or when no intention was stated. Exploratory analyses may suggest that participants were less vigilant in processing the feedback when it was delivered with the intention to help (as indicated by decreased P200 amplitudes). Furthermore, unexpectedly increased P300 and LPP amplitudes in trials where feedback senders communicated their moral superiority than their intention to help could suggest that participants were especially motivated to pay attention to these messages.

## General Discussion

People can be reluctant to listen to outgroup members who voice criticism and they often respond defensively to such criticism (Hornsey et al., 2002; Hornsey & Imani, 2004; Rösler et al., 2021, 2023). In the current research, we investigated how outgroup critics can address three obstacles they face when criticizing others on their morality. The first obstacle relates to people responding defensively when they are criticized on their morality. The second obstacle relates to people assuming that outgroup critics have less constructive intentions than ingroup members. The third obstacle relates to people being inclined to interpret moral criticism as a way for the feedback sender to show off that they are morally superior.

The current research tested whether having outgroup feedback senders communicate their helpful intentions when giving feedback can address these obstacles. Indeed, making intentions explicit spoke to all three obstacles as it a) decreased the gap between assumed intentions of ingroup and outgroup feedback senders, b) decreased perceived unfairness of negative moral feedback, and c) indicates reduced decreased attentional vigilance when cognitively processing feedback messages (i.e., decreased P200 amplitudes). In the following, we discuss these main findings in more detail.

### Making intentions explicit diminishes group effects

In line with the growing literature on the ineffectiveness of criticism delivered by outgroup members (Esposo et al., 2013; Hornsey et al., 2004; Hornsey & Imani, 2004; Rösler et al., 2021, 2023; Thürmer et al., 2019; Thürmer & McCrea, 2018), we conceptually replicate that people assume more negative intent from and are more defensive toward feedback senders who belong to an outgroup than an ingroup. Specifically, in the conditions where intentions were not (yet) made explicit in Study 1, participants assumed worse intent from outgroup than ingroup senders. They also perceived negative feedback on their morality as more unfair if this was communicated by outgroup rather than ingroup senders. While the findings were more robust for assumed intent than for perceived fairness, our studies clearly demonstrate that when senders communicate their reasons behind providing feedback, it reduces the impact of their group membership on recipients’ expectations regarding the sender’s intentions and their evaluations of the feedback. This is a noteworthy finding, as any stated intentions communicated alongside negative moral feedback could also have been questioned or perceived as not credible (especially when stated by an outgroup member), which was not the case.

### Communicating the intention to help decreases perceived unfairness

Across two studies, we show that when feedback senders communicated their intention to help, this made participants perceive negative feedback on their morality as less unfair than when senders did not make their intentions explicit or when they demonstrated their moral superiority. Thus, as an outgroup critic, but also as an ingroup critic, communicating having helpful intentions and not “giving people the space” to make assumptions is an easy and effective way to increase the impact of negative feedback.

The exploratory ERP results of Study 2 uncover potential underlying mechanisms of these changes in self-reported perceived unfairness. Even though not initially predicted, we found that when feedback senders communicated their intention to help alongside negative moral feedback, this seemed to decrease P200 amplitudes compared with trials in which senders did not communicate their intentions or communicated their moral superiority. The functionality of the P200 for word processing (i.e., in our case feedback messages) is less well understood than other ERPs. But, modulations of P200 amplitudes have been associated with the initial lexical encoding of words (Kissler et al., 2006; Trauer, Andersen, Kotz, & Müller, 2011) and in research on the social categorization of faces, larger P200 amplitudes have been associated with “rapidly occurring vigilance” (Ito & Bartholow, 2009). Thus, it is possible that participants were less vigilant toward the processing of negative moral feedback messages when senders communicated their intention to help (vs. their moral superiority or not communicating intentions), indicating that they felt less threatened. It would be interesting to test this effect with future research to be able to draw stronger conclusions.

Against our initial predictions, participants seemed to be more motivated to pay prolonged attention to negative moral feedback messages when senders communicated their moral superiority, as indicated by larger P300 and LPP amplitudes, compared with communicating the intention to help or not communicating intentions. This finding may be explained by considering research on “moral do-gooders” and social comparisons (Minson & Monin, 2011; Wills, 1981). This research has shown that people often get annoyed when others show off their morality to make themselves feel better. More research is needed to confirm these interpretations, but we may conclude that being criticized for one’s morality by someone who has the intention to show off their own morality makes people especially annoyed and agitated. Such messages may hold people’s attention longer and are perceived as more unfair.

Together, these findings suggest that communicating the intention to help when criticizing people on their morality can make receivers perceive the criticism as less unfair, potentially because they feel less threatened. Outgroup critics, as well as ingroup critics, are therefore advised to fill the “gap” people use to make assumptions about the motives of critics by making their helpful intentions explicit.

### Limitations and avenues for future research

Previous experimental research has found that participants are more emotionally affected by receiving negative criticism delivered by ingroup members than outgroup members (Rösler et al., 2023). Even though at first glance this may seem contradictory to the findings of the current research and the literature on the intergroup sensitivity effect (Hornsey et al., 2002), it may be explained by the underlying psychological mechanism elicited by the nature of the experimental designs. In this previous research, participants first underwent the experimental task in which they received positive and negative feedback messages delivered by individual ingroup and outgroup members. Following this task, they received a bogus feedback message from each group, ostensibly averaging all feedback messages delivered in the experimental task and specifying that the feedback was more negative than positive. A negative evaluation from the ingroup elicited a stronger emotional and defensive reaction in participants than when the same message was delivered by the outgroup. This reaction may represent an emotional response, indicating that people care more about being evaluated by ingroup senders than outgroup senders (as also reflected in brain responses, participants were more vigilant when processing ingroup feedback, as indicated by increased P200 amplitudes). The current research, on the other hand, in which we asked participants to rate the fairness of the feedback messages and made the feedback sender’s intentions explicit, might have prompted a more cognitive evaluation of the situation. In the intergroup sensitivity literature, most research concerns such cognitive evaluations of criticism situations rather than receiving criticism from group members in the actual moment (Esposo et al., 2013; Hornsey & Imani, 2004; Sutton et al., 2006). It, therefore, makes sense that in the current research, participants respond more negatively to criticism delivered by outgroup senders than ingroup senders (when intentions are not made explicit).

We defined group memberships as senders having a similar or different SVO-type as the participant. Feedback in the experimental task related to the donation decisions of participants but was allegedly coming from participants’ judgments of a previous experiment where these previous participants judged people who made similar donation decisions as the current participant. Even though we carefully instructed participants to interpret the feedback as personal feedback on their own choices and personality, it is possible that some participants interpreted the feedback as instead referring to their group identity. It would be interesting to test in future research whether our effects replicate using a different group-identity manipulation, for example, ingroups and outgroups at the workplace. In line with previous research using these group identities and showing conceptually similar effects (Rösler et al., 2021), we would expect that they would.

Another intriguing avenue for future research involves investigating whether the intensity of the group manipulation influences the observed effects. Previous studies within the intergroup sensitivity literature have demonstrated that even subtle alterations to group memberships, such as vaccination status and dietary preferences, can trigger significant group-related effects (Thürmer et al., 2022; Thürmer & McCrea, 2022). Our current research likewise employed a relatively subtle group-membership manipulation. It is likely that ingroup/outgroup source effects are amplified when referring to more socially meaningful groups, for instance, based on political affiliations. Following up on the current research, it would be of interest to establish whether explicitly communicating helpful intentions also can mitigate source effects in these other cases.

A limitation of the current research is that the paradigm was unbalanced, meaning that the feedback participants received on their behavior depended on their choices. Even though this was needed (and intended to) increase the relevance and credibility of feedback for participants, it also comes with drawbacks. Participants were not always presented with the same number of trials per condition. In the current research, we, therefore, used linear mixed modeling for the analyses of self-report and EEG data. This method can account for “missing” data. Moreover, we used mean rather than peak amplitudes for the ERP analyses, so a differing number of trials is less influential. However, future research should replicate our findings using a more balanced design.

Another drawback of using an unbalanced design is that we needed to exclude participants from the EEG sample because of not having enough trials left to achieve a good signal-to-noise ratio for each of the three experimental conditions. Even though sensitivity power simulations confirmed that we had enough power to detect ERP effects (see SI), it would still be advisable to replicate the ERP findings with a larger sample.

## Conclusions

The current research offers promising findings with important implications. Rather than letting others make assumptions about one’s intentions when giving feedback, we advise outgroup, as well as ingroup, feedback senders to communicate their intentions and to emphasize their willingness to help. Doing so might prevent defensive reactions from feedback receivers and with that encourage their moral growth.

### Supplementary Information

Below is the link to the electronic supplementary material.

Supplementary file1 (DOCX 1545 KB)

## Footnotes

1 Based on an unexpected finding of a pilot study, we additionally specified a hypothesis in our preregistration about how communicated intentions might affect actual (task-related) behavior. However, we did not find evidence for this in the current studies and therefore did not include it in the paper. Moreover, the preregistration included two studies, each testing a slightly different dependent variable (i.e., perceived fairness of the feedback message and taking the feedback to heart) as we investigated which of these two is more relevant for our current research. Although we found similar results for both studies, we chose perceived fairness as the more relevant dependent variable.

2 Analyses of the money allocation task showed that for Study 1 participants allocated 51% of all possible donations (i.e., contributing the full amount in each trial) and for Study 2 54%.

3 Due to a coding error participants received a little less negative feedback in one of our conditions and instead received positive feedback. Excluding these cases for all three conditions yielded the same results.

4 Additionally, we collapsed the data across different experimental conditions to be able to check the overall interaction between the sender’s group-membership and communicated intentions including the control condition (helping vs. moral superiority intention, which was manipulated within-participants vs. no intention, which was manipulated between-participants). As would be expected, these main effects were qualified by an interaction effect between the sender’s group-membership and the factor type of intention communicated, helping vs. no intentions: *B* = .51, t = 3.14, *p* = .002, 95% CI = [.19, .83], moral superiority vs. no intentions: *B* = .60, t = 3.51, *p* = .001, 95% CI = [.26, .93].

5 Please note that the sample was unbalanced for this analysis, as the control condition included two of the between-participants factors and the intentions condition included only one.

6 We again collapsed the data across conditions to check the interaction effect between the sender’s group-membership and communicated intentions including the control condition. This interaction effect was not significant, *ps* > .111*.* This may be explained by a floor effect, indicating that participants perceived negative moral feedback as quite unfair and making it challenging to detect small variations in the variable. In fact, when log-transforming perceived fairness to account for a floor effect, the interaction effect was significant for the contrast helping vs. no intentions: *B* = .09, *t* = 2.00, *p* = .048, 95% CI = [.0008, .18], but not for moral superiority vs. no intentions: *B* = .04, *p* = .392.

7 Please note that the sample was unbalanced for this analysis, as the control condition included one of the between-participants factors and the intentions condition included two.

8 Besides hypotheses about the P300 and LPP, we had also predicted that people would show enhanced social categorization of outgroup (vs. ingroup) faces as indicated by increased N200 amplitudes when viewing faces (as in previous research, see SI of Rösler et al., 2023). However, we didn’t find any (reliable) effects on social categorization (see SI).

9 Initially, we had planned to test 50 participants, however, due to constraints in lab time and since we already had made our minimum sample we stopped here.
