# Supplementary material for: Beneficial effects of communicating intentions when delivering moral criticism: Cognitive and neural responses
Source: Cogn Affect Behav Neurosci. 2024 Feb 14;24(3):421–39. doi: 10.3758/s13415-024-01164-1 (PMC11078822; doi:10.3758/s13415-024-01164-1)
Supplement: Supplementary file 1 — Supplementary file1 (DOCX 1545 KB) [file 13415_2024_1164_MOESM1_ESM.docx]

**Supplementary Information**

**Study 1**

**Methods**

**Recall task.** To explore whether the factor time of communicated intentions (i.e., before the sender was presented, before the message was presented, and no intentions communicated) would modulate the cognitive processing of feedback messages, we added a recall task of feedback messages at the end of the experiment. Checking whether participants recalled feedback messages more often in one of the conditions, for example, when intentions were communicated right before the feedback message was shown, could indicate whether participants also allocated more attentional resources to process feedback (as an indication of potential ERP effects). We did not find any differences for this task, *F* < 1.

**Results**

**Participants’ SVO types.** 88.6% of the participants classified as a prosocial SVO type, and 11.4% as an individualist SVO type.

**Comparing negative to positive feedback messages**. To get an insight into how participants scored on perceived fairness of moral feedback messages in all trials (i.e., both negative and positive feedback), we averaged the scores across trials. We then computed one-sample t-tests against the midpoint of the two scales (i.e., 4). We checked our anticipated effect of whether negative feedback was perceived as less fair than positive feedback. Indeed, averaged scores of perceived fairness differed significantly from the midpoint of the scale for negative feedback, *M* = 1.96, *SD* = .89, *t*(42) = -14.96, *p* < .001, 95% CI = [1.69, 2.24], indicating that participants generally perceived negative feedback on their morality as not very fair. For positive feedback, they did not differ from the midpoint, *M* = 4.33, *SD* = 1.26, *t*(40) = 1.69, *p* = .099, 95% CI = [3.93, 4.73], indicating that participants perceived positive feedback as not especially fair or unfair. Comparing these two averages with a paired-sample t-test revealed that, as anticipated, negative feedback was perceived as less fair than positive feedback *t*(39) = -10.61, *p* < .001, 95% CI = [-2.71, -1.84].

**Exploratory analyses: Money contributions on subsequent trial.** There were no effects of valence, sender’s group-membership, and type of intention on behavioral decisions on the subsequent trail, *p* > .183

**Study 2**

We additionally investigated social categorization processes of sender faces to check whether participants initially distinguish between ingroup and outgroup faces by preferentially processing faces of ingroup or outgroup members. We predicted that participants would show greater amplitudes on ERPs related to social categorization (i.e., N200) for outgroup as compared to ingroup faces.

**Method**

We matched the feedback sender’s gender to the participant’s gender. We counterbalanced the faces displayed as ingroup members and outgroup members to ensure that face characteristics do not account for any potential social categorization effects. That is, the same set of faces was shown to half of the participants as belonging to the ingroup, whereas for the other half of the participants, it was shown as belonging to the outgroup. This ensured that ERPs related to the perceptual processing of faces (e.g., N170) are only due to the group-membership of the senders, rather than their facial features.

**Perceptual attention and social categorization of faces**

We selected the electrodes to include in the analyses based on the literature and the visual inspection of the grand averages. Consistent with past literature (Ito & Urland, 2003; Van Nunspeet et al., 2014), ERPs related to perceptual attention and social categorization of faces (i.e., N100, P200) were evident at centro-frontal and centro-parietal electrodes Fz, FCz, Cz, CPz, and Pz. Mean amplitudes for the N100 were averaged between 100-125ms, and for the P200, between 140–170ms post stimulus-onset. We could not identify a clear N200 in the current research.

For the N170, consistent with past literature (Ito & Urland, 2003), there was a peak at the left posterior P7 and right posterior site P8. Mean amplitudes for the N170 were averaged between 120–180 ms post stimulus-onset.

**Results**

**Participants’ SVO types.** 85.3% of the participants classified as a prosocial SVO type, and 14.7% as an individualist SVO type.

**Comparing negative to positive feedback messages.** Replicating the results of Study 1, one-sample t-tests against the midpoint of the scales (i.e., 4) showed that averaged scores of perceived fairness were significantly lower from the midpoint of the scale for negative feedback (*M* = 1.88, *SD* = .63), *t*(33) = -19.81, *p* < .001, 95% CI = [1.66, 2.09]. This means that negative feedback was generally perceived as not very fair. Positive feedback was perceived as fair, as indicated by averaged scores of perceived fairness being significantly higher from the midpoint of the scale, (*M* = 4.66, *SD* = 1.47), *t*(31) = 2.53, *p* = .017, 95% CI = [4.13, 5.18]. Both means were also significantly different from each other, *t*(31) = -9.93, *p* < .001, 95% CI = [-3.34, -2.20].

**Perceptual attention and social categorization of faces.** We first tested whether the social group-membership (ingroup vs. outgroup) modulated ERPs related to the perceptual attention and social categorization of faces of feedback senders. We predicted N100 and P200 amplitudes using LMMs (ML) and submitted the participant number as a random effect and the sender’s group-membership (ingroup vs. outgroup) and electrode site (Fz vs. FCz vs. Cz vs. CPz vs. Pz) as fixed effects. For the N170, we used the same model but submitted relevant electrodes for the N170 (i.e., P7 vs. P8). There were no effects of sender’s group-membership on these ERPs, *ps >* .813.

**Exploratory analyses: P3a**. We used an LMM to predict mean P3a-amplitudes (200-300ms) and submitted communicated intentions (helping vs. superiority intention vs. control [no intention communicated]) and electrode site (Pz vs. CPz vs. Cz) as fixed effects to this model. The results showed that when senders communicated their intent to help, this decreased P3a-amplitudes compared to the control condition, *B* = -0.87, *t* = -3.75, *p* < .001, 95% CI = [-1.32, -.42] (see Figure S1), and compared to the moral superiority condition, *B* = -1.09, *t* = -4.34, *p* < .001, 95% CI = [-1.59, -.60]. There was no difference between the latter two conditions, *p* = .337. Because the P3a is associated with expectancy violations (Polich, 2007), these results may suggest that participants were less surprised to see a negative feedback message when senders had communicated their intention to help than when they had communicated their moral superiority or not communicated their intentions. This is in line with the results of the P200, which showed that participants were less vigilant when negative feedback was given with helpful intentions and may indicate that people were more open to receiving negative feedback in these cases.

**Exploratory analyses: Money contributions on subsequent trial.** We find that participants contributed more money when they had received positive feedback in the previous trial (which was given because they had contributed most of the money to the charity), *B* = .62, *t* = 3.37, *p* < .001, 95% CI = [.25, .98], rather than when they had received negative feedback for a selfish decision in the previous trial. Interestingly, for negative feedback trials, there was also an interaction effect between the sender’s group-membership and the type of intentions senders communicated (see Figure S2) (comparison helping vs. control: *B* = 1.21, *t* = 3.22, *p* = .001, 95% CI = [.26, .98]). Simple effects showed that compared to communicating no intentions, when outgroup members communicated the intention to help, this motivated participants to give more money in the following trials, *B* = 1.31, *t* = .27, *p* < .001, 95% CI = [.78, 1.84]. For ingroup members, there was no significant difference, *p* = .549. This suggests that, as an outgroup member, communicating the intention to help is an especially useful strategy to encourage behavior change. This effect may be due to an expectancy violation. Since people expect more negative intentions from outgroup members and more positive intentions from ingroup members, they may ‘reward’ outgroup members for communicating helping intentions.

**Tables**

**Table S1**

*List of Sentences Used for Intentions Manipulation (in Dutch as Used in Study)*

| Acknowledgment | Helping | Moral superiority |
| --- | --- | --- |
| Ik zie dat je het belangrijk vindt om geld te geven aan goede doelen | Ik wil je helpen om overeenkomstig te handelen met jouw eigen waarden | Ik geef altijd meer geld aan goede doelen vergeleken met jou |
| Het is geweldig dat je geeft om doneren aan goede doelen | Ik wil je helpen jouw doel te bereiken als het gaat om doneren aan goede doelen | Ik ben een beter persoon dan jij, omdat ik meer geld geef aan goede doelen |
| Je lijkt het belangrijk te vinden om te geven aan mensen in nood | Ik wil je herinneren dat je hebt gezegd dat we geld zouden moeten geven aan goede doelen zodat je overeenkomstig met jouw waarden kunt handelen | Ik wil je met dit oordeel laten zien dat ik een beter persoon lijk te zijn dan jij |
| Fijn om te zien dat je erom geeft om anderen te helpen | Je zei dat het belangrijk was om geld te geven aan goede doelen en ik wil je helpen overeenkomstig te handelen met jouw waarden | Ik wil duidelijk maken dat ik een beter persoon ben dan jij |
| Het lijkt alsof het belangrijk is voor jou om te doneren aan goede doelen | Ik wil je helpen door je te herinneren dat je hebt gezegd dat het belangrijk is om te doneren aan goede doelen | Ik lijk een beter persoon te zijn dan jij en daarom geef ik je dit oordeel |
| Je laat zien dat je het op waarde stelt om mensen in nood te helpen |  | |
| Ik zie dat je geld hebt gegeven aan dit goede doel, dat is fijn |  |  |
| Geven aan mensen lijkt iets te zijn wat jij op waarde stelt |  |  |
| Het is fijn dat je geeft aan mensen in nood |  | |
| Wat fijn dat je geld hebt gegeven aan dit goede doel |  | |

**Table S2**

*List of Sentences Used for Intentions Manipulation (Translated to English)*

| Acknowledgment | Helping | Moral superiority |
| --- | --- | --- |
| I see that it is important to you to give money to charities | I want to help you act in line with your own values | I always give more money to charities compared to you |
| It’s great that you care about giving to charity | I want to help you achieve your goal to give to charities | I'm a better person than you because I give more money to charities |
| You seem to care about giving to people in need | I want to remind you that you said we should give money to charities so you can act in line with your values | I want to show you with this judgment that I seem to be a better person than you |
| Nice to see that you care about helping others | You said it was important to give money to charities and I want to help you to act in line with your values | I want to make it clear that I am a better person than you |
| It seems like giving to charities is important to you | I want to help you by reminding you that you said you think it is important to give to charities | I seem to be a better person than you and that’s why I give this judgment |
| You show that you value to help people in need |  | |
| I see that you gave money to this charity, that’s nice |  |  |
| Giving to people seems to be something you value |  |  |
| It’s nice that you give to people in need |  | |
| How nice that you gave money to this charity |  | |

**Figures**

**Figure S1**

*Raincloud Plots for the Effects of Communicated Intentions (i.e., Helping vs. Moral Superiority Intentions vs. No Intentions) on P3a-amplitudes*


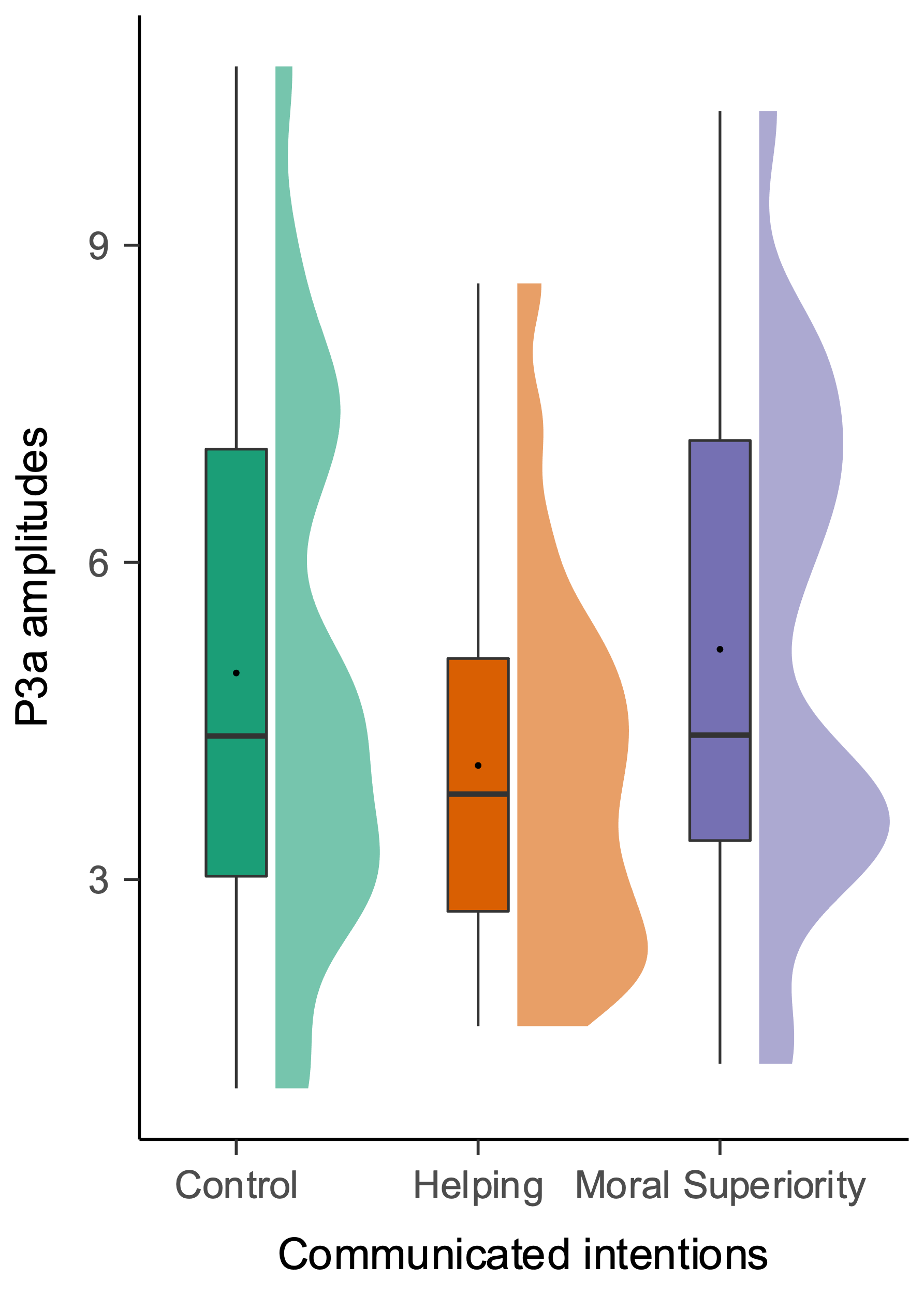


**Figure S2**

*Money Contributions in Next Trial per Sender’s Group-membership and Intention Communicated (Study 2)*


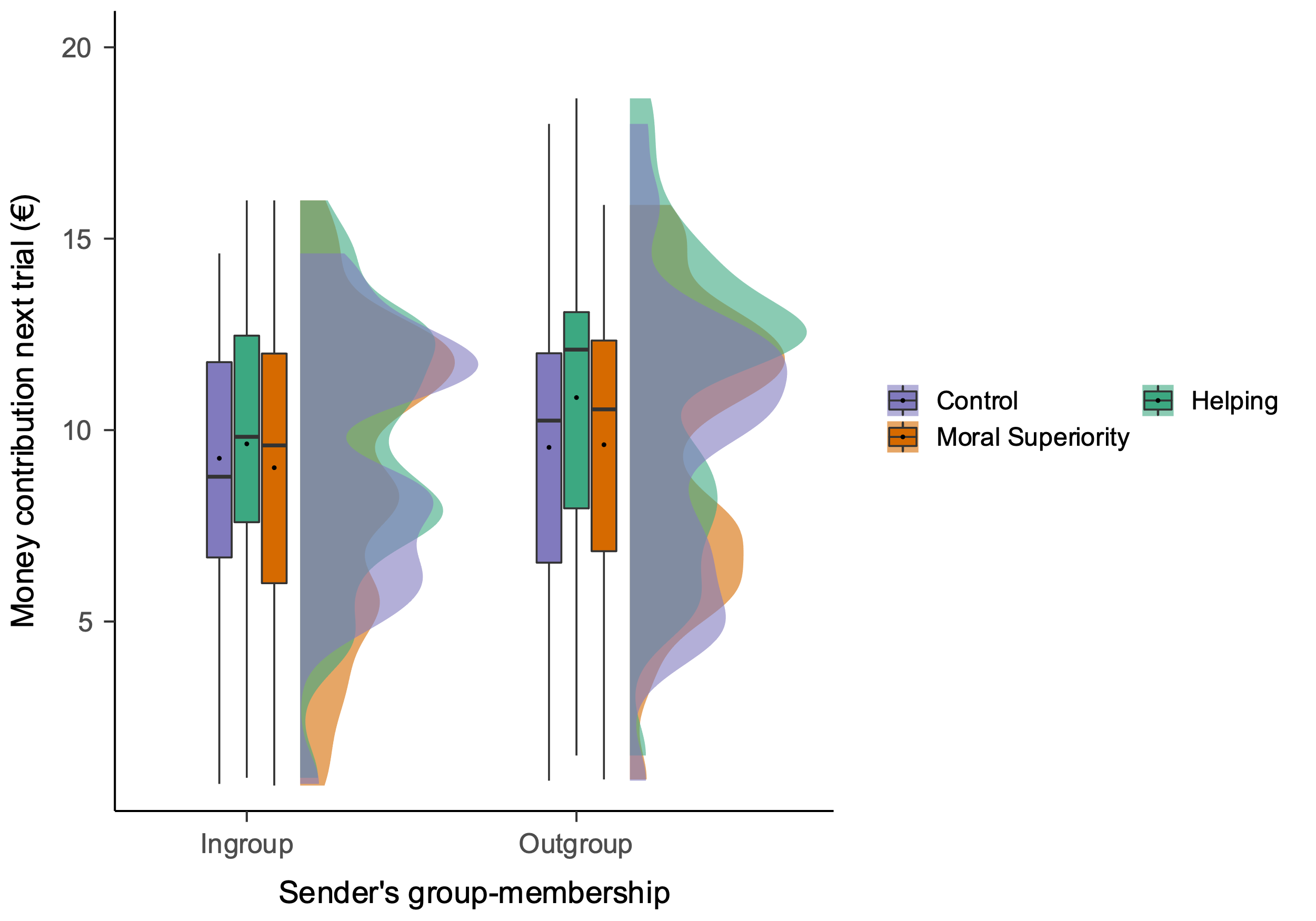


**Figure S3**

*ERP plots Displaying Grand Averages and Standard Deviations*

**Figure S4**

*Raincloud Plots for the Effects of Communicated Intentions (i.e., Helping vs. Moral Superiority Intentions vs. No Intentions) on Early LPP-amplitudes*

*
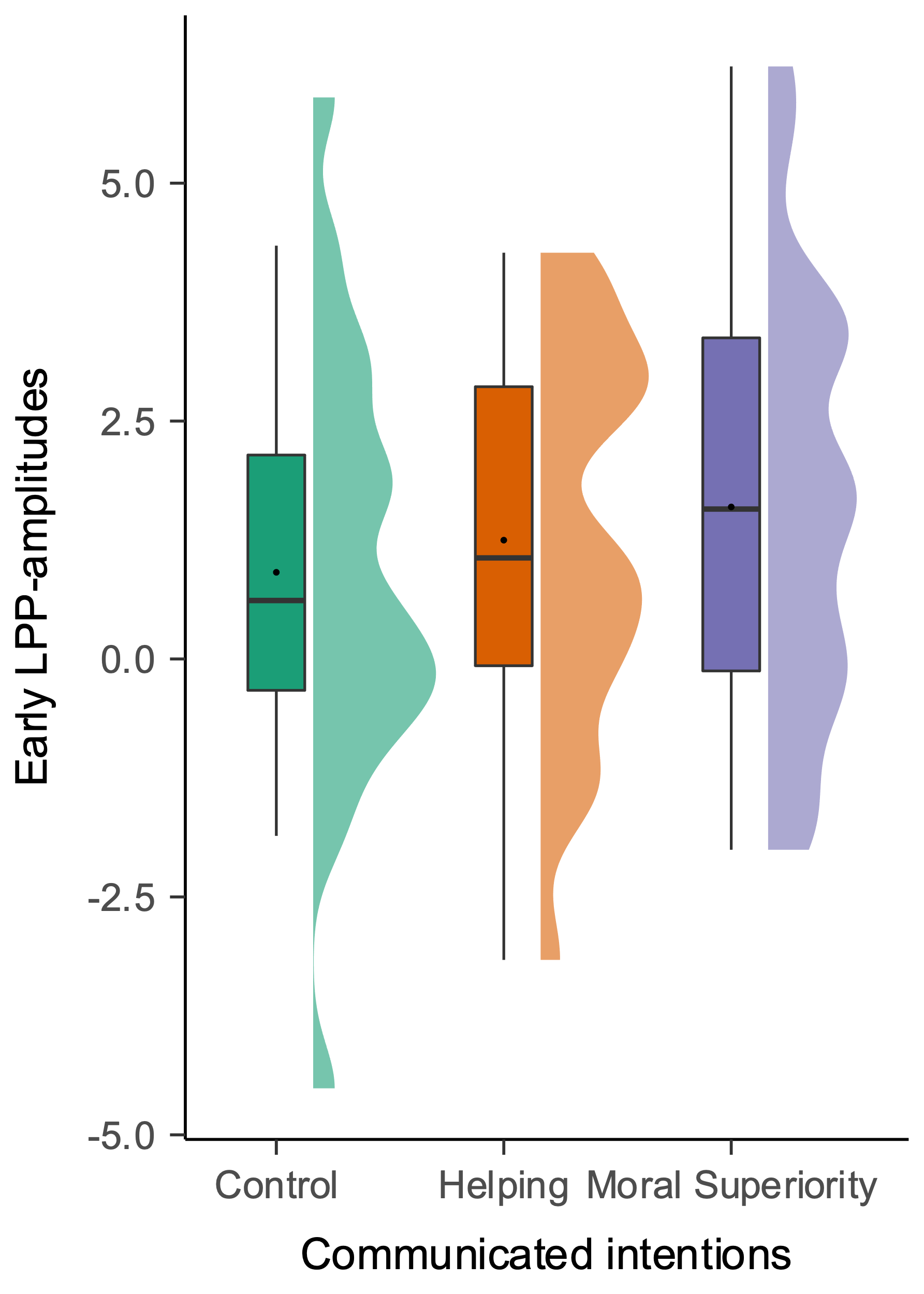
*

**Sensitivity Power Simulations** (across studies)

When planning our research, we performed power calculations based on previous research that investigated whether feedback on one’s competence is more effective than on one’s morality by asking participants to reflect on criticism situations at their workplace (Rösler et al., 2021). We deemed this prior research as relevant to the current research, as it investigated a strategy to increase the impact of feedback, similar to the aim of the current research. We used sample size calculation for repeated measure designs in G*power as a proxy for linear mixed models. The effect size of the strategy, the social dimension addressed in the negative feedback message (i.e., morality vs. competence), on the acceptance of negative feedback (see footnote in Rösler et al., 2021) was η^2^_p_ = .06 (this corresponds to a mean difference of 0.56 on a 7-point Likert scale).

After completing our study, we shifted our approach to sensitivity power simulations, as advocated by DeBruine and Barr (2021). This analysis more directly tests power for mixed models (instead of using sample size calculation for repeated measures as an indicator) and can give insights into whether our studies had enough power to detect the observed effects. We extracted relevant parameters from our analyses and used these as data-generating parameters for the simulations. Because the current research used an unbalanced design that was based on participants’ choices, we used the average trial number for the behavioral data and the minimum trial number for the EEG data.

The results, displayed in Figure S5, show that assuming 80% power, for the effect of senders’ group-membership on assumed intentions, we can detect effect sizes as small as *B* = 0.3. The actual effect size, in this case indicating the mean difference on the 7-point Likert scale, was *B* = 0.77, indicating that we have enough power to detect this effect. It is slightly lower than the mean difference found in previous research (i.e., 0.92, Rösler et al., 2021). For the effect of having senders communicate helping versus moral superiority intentions, we can detect effect sizes as small as *B* = 0.6 for perceived fairness in Study 1, *B* = 0.2 for perceived fairness in Study 2, and *B* = 1.0 for the P200 in Study 2 (see Figure S6). The effect sizes extracted from our analyses are well above these thresholds for the P200 (i.e., *B* = 1.38, this was an unexpected finding). For perceived fairness, however, the observed effect sizes are smaller than initially expected (expected: 0.56, Rösler et al., 2021, found: Study 1: *B* = 0.25, Study 2: *B* = 0.10), and fall below the threshold. This may be attributed to a floor effect that emerged due to our focus on negative moral feedback in the current research. It may reflect that the distribution of the observed effects is skewed, therefore reducing the effect size (also see footnote 5 in the manuscript). Since the effect was preregistered and emerged in both studies, we can be somewhat confident that this effect is indeed caused by our manipulation, rather than due to a measurement error.

**Figure S5**

*Sensitivity Power Simulations for the Effect of Ingroup vs. Outgroup Sender on Assumed Intentions*


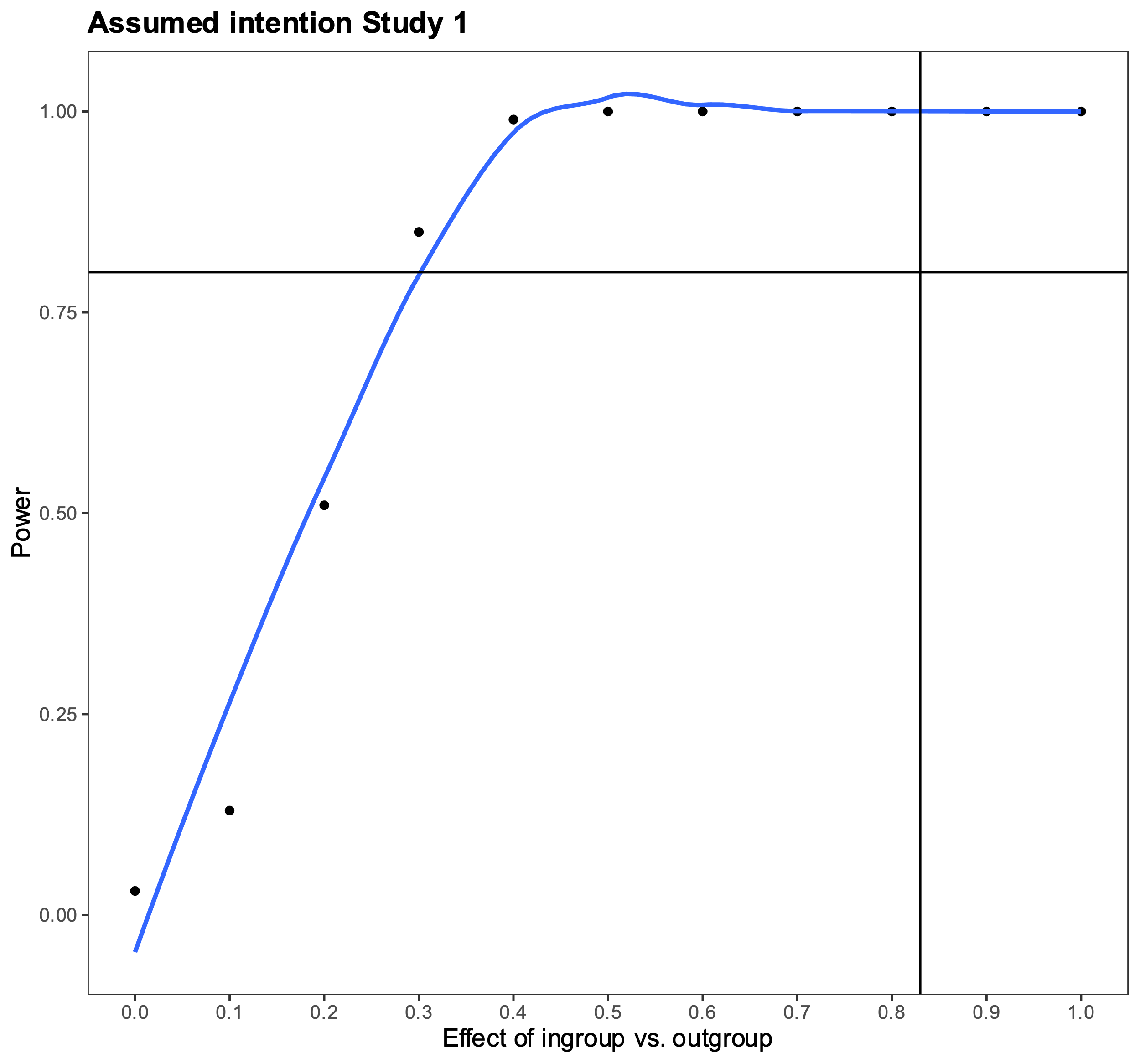


**Figure S6**

*Sensitivity Power Simulations for the Effect of Helping vs. Moral Superiority Intentions on Perceived Fairness and P200*

**Reliability of measures** (across studies)

To check whether our measures are reliable, we ran two separate additional analyses. We first checked reliability by testing whether our effects were observed across different trials. We added the trial number to the models of our main analyses as an indicator of time. The rationale underlying this approach was that if our measures are reliable, the trial number should not interact with our effects of interest. Second, we examined the variance explained by clusters in our data—calculating the intraclass correlation coefficients (ICC) for trial number and intention manipulation sentences, when adding these to the models. Here, high reliability would be indicated by a small percentage of variance explained by these two clusters, as the ICC describes how strongly values in a cluster resemble each other.

For Study 1, both for effect of sender’s group-membership on assumed intentions and the effect of type of communicated intentions on perceived fairness, the trial number showed a significant main effect such that participants selected slightly lower scores towards the end of the experiment, group: *B* = -.02, *t* = -3.88, *p* < .001, intentions: *B* = -.01, *t* = -2.31, *p* = .021. Importantly, trial number neither interacted with the effect of sender’s group-membership on assumed intentions, *p* = .178, nor with the effect of communicated intentions on perceived fairness, *p* = .873. ICCs for trial number and intention manipulation sentences were below .001. Thus, we may assume that our measures were reliable.

For perceived fairness of Study 2, there was no main effect of trial number, *p* = .076. There were interaction effects between the trial number and helping intentions (vs. control), *B* = -.001, *t* = -2.07, *p* = .039, and helping intentions (vs. moral superiority intentions), *B* = -.002, *t* = -2.37, *p* = .018. However, these effects were very small (< .002) and do not indicate great fluctuation of our measures over time. ICCs for trial number and intention manipulation sentences were below .021. Thus, we may assume that our behavioral measures were reliable.

Given that we used aggregated trial data for the EEG measures in Study 2, we were unable to examine the effects of trial number on ERPs. However, we ensured the reliability of measures by planning and preregistering the number of trials needed for a sufficient signal-to-noise ratio. That is, for each participant, there was a minimum trial number of 30 trials in each of our conditions (Luck, 2005). Thus, we may assume that our EEG measures are reliable.

References

DeBruine, L. M., & Barr, D. J. (2021). Understanding Mixed-Effects Models Through Data Simulation. *Advances in Methods and Practices in Psychological Science*, *4*(1). https://doi.org/10.1177/2515245920965119

Ito, T. A., & Urland, G. R. (2003). Race and Gender on the Brain: Electrocortical Measures of Attention to the Race and Gender of Multiply Categorizable Individuals. *Journal of Personality and Social Psychology*, *85*(4), 616–626. https://doi.org/10.1037/0022-3514.85.4.616

Luck, S. J. (2005). *An Introduction to the Event-Related Potential Technique*. MIT Press.

Polich, J. (2007). Updating P300: An Integrative Theory of P3a and P3b John. *Clin Neurophysiol.*, *118*(10). https://doi.org/10.1016/j.clinph.2007.04.019.Updating

Rösler, I. K., Van Nunspeet, F., & Ellemers, N. (2023). Falling on death ears: The effects of sender identity and feedback dimension on how people process and respond to negative feedback − an ERP study. *Journal of Experimental Social Psychology*, *104*.

Van Nunspeet, F., Ellemers, N., Derks, B., & Nieuwenhuis, S. (2014). Moral concerns increase attention and response monitoring during IAT performance: ERP evidence. *Social Cognitive and Affective Neuroscience*, *9*(2), 141–149. https://doi.org/10.1093/scan/nss118
